# Weight Loss Outcomes Among Early High Responders to Exenatide Treatment: A Randomized, Placebo Controlled Study in Overweight and Obese Women

**DOI:** 10.3389/fendo.2021.742873

**Published:** 2021-11-17

**Authors:** Megan Rodgers, Alexandra L. Migdal, Tahereh Ghorbani Rodríguez, Zsu-Zsu Chen, Anjali K. Nath, Robert E. Gerszten, Natasha Kasid, Elena Toschi, Juliet Tripaldi, Brent Heineman, Minh Phan, Long Ngo, Eleftheria Maratos-Flier, Jody Dushay

**Affiliations:** Beth Israel Deaconess Medical Center, Boston, MA, United States

**Keywords:** overweight, obesity, weight loss, exenatide, hypocaloric diet

## Abstract

**Objective:**

As there is significant heterogeneity in the weight loss response to pharmacotherapy, one of the most important clinical questions in obesity medicine is how to predict an individual’s response to pharmacotherapy. The present study examines patterns of weight loss among overweight and obese women who demonstrated early robust response to twice daily exenatide treatment compared to those treated with hypocaloric diet and matched placebo injections.

**Methods:**

We randomized 182 women (BMI 25-48 kg/m2) to treatment with exenatide alone or matched placebo injections plus hypocaloric diet. In both treatment groups, women who demonstrated ≥ 5% weight loss at 12 weeks were characterized as high responders and those who lost ≥10% of body weight were classified as super responders. Our primary outcome was long-term change in body weight among early high responders to either treatment. An exploratory metabolomic analysis was also performed.

**Results:**

We observed individual variability in weight loss with both exenatide and hypocaloric diet plus placebo injections. There was a trend toward a higher percentage of subjects who achieved ≥ 5% weight loss with exenatide compared to diet (56% of those treated with exenatide, 76% of those treated with diet, p = 0.05) but no significant difference in those who achieved ≥ 10% weight loss (23% of individuals treated with exenatide and 36% of those treated with diet, p = 0.55). In both treatment groups, higher weight loss at 3 months of treatment predicted super responder status (diet p=0.0098, exenatide p=0.0080). Both treatment groups also demonstrated similar peak weight loss during the study period. We observed lower cysteine concentrations in the exenatide responder group (0.81 *vs* 0.48 p < 0.0001) and a trend toward higher levels of serotonin, aminoisobutyric acid, anandamide, and sarcosine in the exenatide super responder group.

**Conclusion:**

In a population of early high responders, longer term weight loss with exenatide treatment is similar to that achieved with a hypocaloric diet.

**Clinical Trial Registration:**

www.clinicaltrialsgov, identifier NCT01590433.

## Introduction

The growing obesity epidemic and its associated co-morbidities, including but not limited to type 2 diabetes and cardiovascular disease, has led to an urgent need for effective treatment options. Recent figures from the Center for Disease Control (CDC) report the prevalence of obesity among adults in the US at 39.8% in 2017 (1). While individuals with obesity can achieve robust weight loss with structured interventional and behavioral programs, weight regain is very common (2, 3). A recent meta-analysis showed that people who lost, on average, 9.9 kg body weight through diet alone, or through a combined diet and exercise regimen, regained approximately 50% of the weight within one year (4). Bariatric surgery is often associated with significant long-term weight loss; however due to strict eligibility criteria, limited insurance coverage, and other personal reasons, many individuals opt against pursuing surgical treatment (5, 6). Investigating weight loss response to pharmacologic agents, including factors that may contribute to heterogeneity of response, is an area of high clinical and public health significance.

One pharmaceutical class generating great interest in this field are the glucagon like peptide 1 receptor agonists (GLP1RA) including exenatide, liraglutide, dulaglutide and semaglutide. Exenatide, the first approved GLP1RA, was developed for the treatment of type 2 diabetes (7). Subsequent studies in individuals without diabetes demonstrate that exenatide, liraglutide and semaglutide are associated with significant weight loss and improvement in metabolic parameters (8–14). A recent review noted that treatment with liraglutide, the first GLP1RA approved by the FDA specifically for weight loss, leads to 5-10% reduction of body weight (8–10). Semaglutide, the most recently approved once-weekly GLP1RA, has been associated with even more weight loss, up to 14% at higher doses, in individuals with obesity (13–15).

We have previously reported variable weight loss in women with overweight or obesity treated in a crossover study with twice-daily exenatide or placebo for 16 weeks; in that study about one-third of participants achieved no weight loss, one-third achieved 3-5% weight loss and one third achieved > 5% weight loss (11). Reasons for this heterogeneity in response to pharmacotherapy are poorly understood. Early weight loss of >4% body weight with liraglutide has been shown to predict 1-year weight loss, but additional factors that predict or account for variability of weight loss achieved with GLP1RA are not well understood (12, 16). The present study examines long-term weight loss among women with overweight and obesity who demonstrate initial robust weight loss with twice-daily exenatide treatment; the primary outcome measure is change in body weight. Secondary objectives include changes in metabolic characteristics, including waist circumference, lipid parameters, resting energy expenditure, and body composition, as well as identification of metabolic characteristics that may predict robust weight loss response. In an exploratory analysis, we sought to identify metabolites that were associated with response to exenatide or diet/placebo.

## Materials and Methods

All study procedures involving human participants were reviewed and approved by the Beth Israel Deaconess Medical Center (BIDMC) Institutional Review Board (ClinicalTrials.gov Identifier: NCT01590433, BIDMC Protocol 2011P000310). Participants were recruited through local print advertisements and online advertisements. All participants provided written informed consent. Study visits were conducted at the Harvard Catalyst Clinical Research Center at BIDMC in Boston, MA in accordance with the Declaration of Helsinki.

Two hundred and forty-nine women were screened and 182 enrolled in a 52-week single-blind interventional trial (**Figure S1**, flow diagram). All subjects were blinded to study treatment. Participants were randomized 3:2 to treatment with either exenatide (n=127) titrated to maximum dosing of 10mcg daily or hypocaloric diet with placebo injections (n=55, hereafter referred to as the diet group). There was no run-in period. Eligibility criteria included age 18-70, BMI 25.0 - 48 kg/m^2^ and no acute or poorly controlled chronic medical problems. Diagnosis of diabetes, history of pancreatitis, pregnancy, lactation, and previous use of exenatide were major exclusion criteria. All subjects self-administered twice daily subcutaneous injections of either exenatide or matched placebo. Subjects in the diet group only received nutrition counseling at every study visit, which included recommendations for a hypocaloric diet (500 kcal/day reduction, with total caloric requirement determined from measured resting metabolic rate). Neither group received counseling about exercise, other than being instructed to maintain their current routine throughout study participation. Study visits took place every 2 weeks for the first month and then every 4 weeks thereafter. Subjects were included in the data analysis if they completed at least 12 weeks of treatment (exenatide n=75, diet n=33).

At 12 weeks, subjects in both groups who had lost ≥ 5% of their body weight were categorized as high responders and continued participating in the study for up to 52 weeks. Those who did not achieve 5% weight loss were categorized as low responders and per protocol design their participation ended. Participants in both groups who lost ≥10% body weight at any point during the study were classified as “super responders”.

Body weight, waist circumference, vital signs and body composition (measured using bioelectrical impedance analysis, Quantum II, RJL Systems) were assessed at each visit by study staff who were blinded to study treatment. Comprehensive metabolic visits occurred after an overnight fast at weeks 0, 12, 24, and 52 and included a blood draw for hematology, electrolyte, and lipid panels, measurement of resting metabolic rate using the Sensormedics Vmax Encore 29 indirect calorimeter (CareFusion,Respiratory Care Inc.,Yorba Linda, CA), and assessment of the thermic effect of food with a liquid meal tolerance test (Boost, 253 kcal, 6.3g fat, 34.8g carbohydrate, 15.8g protein). Blood was drawn 1, 2, 3 and 4 hours following ingestion of Boost for measurement of postprandial triglyceride and insulin levels.

In an exploratory analysis, we sought to identify metabolites that were associated with treatment response by leveraging a targeted liquid chromatography-mass spectrometry (LC-MS) method that measures ~150 known metabolites (14). This platform measures polar metabolites that fall into 8 classes: 1) amines; 2) amino acids and amino acid conjugates; 3) bile acids; 4) sugars and sugar phosphates; 5) indoles and indole derivatives; 6) organic acids; 7) purines and pyrimidines; and 8) acylcarnitines. In addition, the platform measures a total of 19 acylcarnitines spanning distinct fatty acid species that contain short-, medium-, and long-carbon chains. We measured these metabolites in a subset of subjects who received exenatide (36 high responders and 28 low responders) and a subset of diet responders (n=23). Metabolomic analysis was done using hydrophilic interaction liquid chromatography (HILIC) coupled with multiple reaction monitoring-based mass spectrometry to measure ~150 endogenous metabolites. Metabolites were extracted from 10 µl of plasma using acetonitrile and methanol. Samples were spiked with deuterated internal standards for quality control. The extracts were separated using reverse-phase chromatography and detected by a coupled 4000 QTRAP mass spectrometer in positive mode (Sciex). In addition to sample injections, control pooled plasma samples were included and spaced every 10 sample injections to gauge the effectiveness of normalization, to adjust for temporal drift of the instrument, and to calculate the coefficient of variation for each metabolite. Metabolite quantification was determined by integrating peak areas using MultiQuant software (Sciex).

### Sample Size Calculation

Based on published literature describing weight loss with exenatide treatment among individuals with type 2 diabetes, we hypothesized that responders would stratify into a plateau group and a continued weight loss group by 52 weeks of treatment. Our sample size was based on a predicted weight loss difference of 1kg between treatment groups and a standard deviation of the distribution of the weight loss difference of 1.2 kg. Assuming 75% of subjects would complete the study until week 12, and 30% would be high responders, and accounting for an additional 25% dropout rate following 12 weeks, we required 38 subjects in the exenatide group and 18 in the diet/placebo group. Given this sample size, we would have a power of 0.83 to detect the difference of 1kg.

The dropout rate from randomization to week 12 was higher than we expected [40% in both the exenatide and diet groups (**Figures S1**, **S2**)], which prolonged the expected enrollment period. However, we observed a lower than expected dropout rate after week 12 (**Figure S2**) and a higher than predicted responder rate in both groups. Owing to funding limitations, we performed data analysis at week 24 with a sample size sufficient to satisfy our initial power calculation.

### Data Analysis

When observing the p-values associated with various variables and group labels, chi-squared modeling and t-tests were conducted to look at parametric testing and non-parametric testing. As the data did not always follow a normal distribution, both methodologies were used to compare the splay of that data, with the reporting value being taken from the chi-squared test. The data used to compare exenatide and diet high responders were taken longitudinally from baseline until the cut-off visit at week 12. Groups were then compared to determine if there were any significant parameters that could predict high responder or super responder status within the treatment groups. The Kruskal Wallis test was used in the models investigating predictors of weight loss in either treatment group. Results are reported as adjusted odds ratios.

We used multivariable logistic regression to assess the weight change (percent change from baseline to 3 months of treatment) as a predictor of weight loss (age, weight, total cholesterol, waist circumference and resting energy expenditure by treatment group). For the analysis of identifying potential physiologic predictors of super responder status, we used the following baseline variables: age (years), weight (kg), BMI (kg/m2), total cholesterol (mg/dL), resting energy expenditure (kcal) and waist circumference (cm).

For the metabolomic data analysis, mean CVs for analytes measured were ≤15%, and closer to 6% for abundant analytes such as amino acids. Metabolites with CVs ≥ 30% were excluded from analysis (15). Ninety-two baseline fasting plasma samples were studied from the two treatment groups (exenatide n = 64, diet/placebo n = 23). Metabolite concentrations determined by LC-MS peak intensities that were normalized using log transformation. A majority of metabolite concentrations were successfully normalized, however, several continued to have non-normal peak distributions as determined by the Shapiro-Wilk test. Wilcoxon-rank sum tests were used to compare baseline metabolite concentrations between responders in the exenatide and diet treatment groups as well as responders verses non-responders, and super-responders verses responders within each treatment group. (www.r-project.org).

## Results

Baseline demographics of the study population are summarized in **Table 1**. There were no significant differences in baseline parameters between the exenatide and the diet groups, nor were there differences in baseline characteristics when comparing the responder populations (**Table S1**). Our study population by ethnicity was 0.5% Native American, 0.5% Native Hawaiian, 1.6% Asian, 24.7% Black or African American, and 73% Caucasian.

**Table 1:** Baseline characteristics of the study population.

	Exenatide (n-75)	Diet (n=33)	*p*
Age (years)	43.9 ± 11.9	44.6 ± 13.7	0.78
BMI (kg/m')	36.1 ± 5.3	34.8 ± 5.0	0.21
Weight (kg)	95.9 ± 14.6	94.0 ± 13.1	0.51
Systolic BP (mmHg)	125.1 ± 12.6	121.2 ± 14.4	0.16
Diastolic BP (mmHg)	73.6 ± 9.3	71.8 ± 12.0	0.40
Heart Rate (bpm)	67.9 ± 10.6	66.1 ± 10.8	0.41
Total Cholesterol (mg/dL)	193.2 ± 36.1	194.7 ± 32.9	0.84
HDL Cholesterol (mg/dL)	59.6 ± 16.8	60.3 ± 11.0	0.83
LDL Cholesterol (mg/dL)	113.0 ± 31.0	109.6 ± 29.8	0.61
Triglycerides (mg/dL)	105.7 ± 52.2	109.4 ± 54.1	0.74
Hemoglobin Ale(%)	5.6 ± 0.3	5.6 ± 0.3	0.33
Waist Circumference (em)	112.1 ± 17.5	10.7 ± 11.9	0.70
Resting Energy Expenditure (kCal/d)	1552.1 ± 202.6	1522.9 ± 199.9	0.49
Percent Body Fat(%)	43.6 ± 4.9	43.7 ± 4.1	0.84

Values are shown as mean +/- SD.

### Weight Loss and Regain Among High Responders and Super High Responders

**Figure 1:**
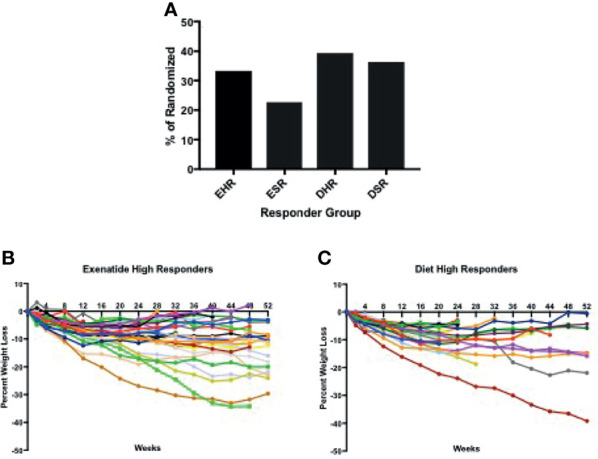
**(A)** Percentage of high and super responders among those randomized to exenatide and diet groups. **(B, C)** Individual weight trajectories in exenatide high responder group **(B)** and diet high responder group **(C)**. EHR exenatide high responder. ESR exenatide super responder. DHR diet high responder. DSR diet super responder.

**Figure 2:**
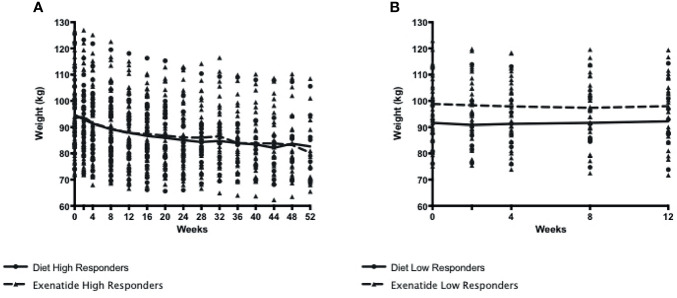
**(A)**. Weight loss among exenatide and diet high responders over 52 weeks **(B)** Weight loss among exenatide and diet low responders over 12 weeks. By protocol design, low responders ended study participation at 12 weeks.

In total, 56% percent of the exenatide group and 76% of the diet group were categorized as initial high responders (p = 0.05, **Figure 1A**). Individual weight loss trajectories were highly variable in both exenatide and diet high responder groups (**Figures 1B, C**). **Figure 2** shows individual and average weight loss among the high and low responder groups over the duration of the treatment period. After 12 weeks of treatment, all groups except diet nonresponders had statistically significant weight loss (**Figure 2** and **Table 2**). Subjects in the exenatide high responder group had an average weight reduction of 6.2 kg and BMI reduction of 2.3 kg/m^2^, while exenatide low responders achieved an average weight reduction of 1.9 kg and BMI reduction of 0.7 kg/m^2^ (**Table 2**). Diet treatment was associated with a 7.2 kg reduction in weight and a BMI reduction of 2.9 kg/m^2^ in the high responder group, while those in the low responder group had a nonsignificant increase in weight (+ 0.6kg) and BMI (+ 0.2kg, **Table 2**).

**Table 2:** Characteristics of all 4 study groups before and after 12 weeks of treatment with exenatide or diet.

	Exenatide High Responders (n = 42)			Exenatide Low responders (n = 33)		
	Week 0	Week 12	p	Week 0	Week 12	p
**BMI (kg/m^2^)**	35.8 ± 5.2	33.5 ± 4.7	<0.0005*	36.5 ± 5.0	35.8 ± 5.0	0.0008*
**Weight (kg)**	93.9 ± 14.9	87.7 ± 13.7	<0.0005*	98.5 ± 13.9	96.6 ± 13.6	<0.0005*
**Systolic BP (mmHg)**	122.1 ± 12.6	119.0 ± 12.6	0.12	124.7 ± 15.5	124.4 ± 15.8	0.90
**Diastolic BP (mmHg)**	69.5 ± 9.3	72.6 ± 10.9	0.08	72.8 ± 9.3	75.0 ± 13.7	0.30
**Heart Rate (bpm)**	68.1 ± 11.3	68.9 ± 11.7	0.58	68.0 ± 8.8	72.8 ± 9.7	0.02*
**Total Cholesterol (mg/dL)**	196.8 ± 34.3	177.9 ± 30.1	<0.0005*	188.4 ± 38.9	180.0 ± 35.6	0.01*
**HDL Cholesterol (mg/dL)**	61.0 ± 19.3	52.9 ± 15.9	0.0005*	55.8 ± 12.0	53.9 ± 11.2	0.20
**LDL Cholesterol (mg/dL)**	115.6 ± 30.8	106.1 ± 27.1	0.0045*	110.8 ± 32.5	105.0 ± 28.9	0.04*
**Triglycerides (mg/dL)**	111.0 ± 61.5	94.1 ± 42.6	0.03*	100.4 ± 37.8	101.2 ± 52.9	0.89
**Insulin (mIU/mL)**	12.2 ± 7.0	11.9 ± 8.8	0.90	11.0 ± 6.5	10.4 ± 4.9	0.43
**Waist Circumference (cm)**	111.8 ± 10.7	105.0 ± 9.5	<0.0005*	111.6 ± 11.9	110.6 ± 11.6	0.54
**Resting Energy Expenditure (kCal/d)**	1548.9 ± 224.4	1411.0 ± 184.5	0.0001*	1556.3 ± 174.3	1560.2 ± 182.5	0.85
**Percent Body Fat (%)**	43.7 ± 4.1	43.3 ± 4.3	0.49	43.8 ± 4.6	43.6 ± 4.1	0.74
**Thermic Effect of Food**	4.4 ± 2.0	3.5 ± 2.4	0.04*	4.3 ± 2.2	2.4 ± 2.8	0.006*
	**Diet High Responders (n = 25)**			**Diet Low Responders (n = 8)**		
	**Week 0**	**Week 12**	**p**	**Week 0**	**Week 12**	**p**
**BMI (kg/m^2^)**	35.1 ± 5.2	32.2 ± 4.3	<0.0005*	34.4 ± 4.5	34.6 ± 4.6	0.67
**Weight (kg)**	94.7 ± 13.6	87.5 ± 11.9	<0.0005*	91.6 ± 11.9	92.2 ± 12.2	0.55
**Systolic BP (mmHg)**	126.0 ± 11.8	117.6 ± 14.0	0.002*	117.1 ± 15.3	114.4 ± 10.5	0.33
**Diastolic BP (mmHg)**	71.7 ± 9.3	69.7 ± 8.6	0.35	67.4 ± 5.8	65.8 ± 9.8	0.46
**Heart Rate (bpm)**	68.1 ± 9.1	66.6 ± 9.8	0.50	69.6 ± 6.1	71.3 ± 7.4	0.65
**Total Cholesterol (mg/dL)**	193.5 ± 28.0	180.1 ± 38.7	0.01*	183.9 ± 39.0	172.9 ± 26.2	0.28
**HDL Cholesterol (mg/dL)**	59.8 ± 11.3	52.5 ± 12.4	0.0003*	61.4 ± 12.4	61.1 ± 17.4	0.95
**LDL Cholesterol (mg/dL)**	113.0 ± 25.6	111.3 ± 34.2	0.64	101.7 ± 27.7	94.4 ± 26.5	0.51
**Triglycerides (mg/dL)**	103.2 ± 47.1	82.4 ± 34.3	0.002*	102.6 ± 57.8	85.7 ± 49.1	0.03
**Insulin (mIU/mL)**	11.4 ± 9.9	7.5 ± 5.3	0.03*	10.4 ± 4.8	11.1 ± 7.0	0.75
**Waist Circumference (cm)**	111.6 ± 11.30	102.7 ± 17.6	0.004*	106.8 ± 10.7	108.9 ± 10.4	0.16
**Resting Energy Expenditure (kCal/d)**	1542.5 ± 194.6	1386.6 ± 116.9	0.0004*	1463.9 ± 217.5	1447.9 ± 248.9	0.78
**Percent Body Fat (%)**	43.8 ± 3.8	42.0 ± 4.6	0.02*	43.7 ± 5.2	43.0 ± 5.1	0.39
**Thermic Effect of Food**	4.5 ± 2.0	4.1 ± 2.0	0.56	4.2 ± 2.0	4.2 ± 2.2	0.96

Values are mean +/- SD. *p values < 0.05 are considered significant.

**Figure 3:**
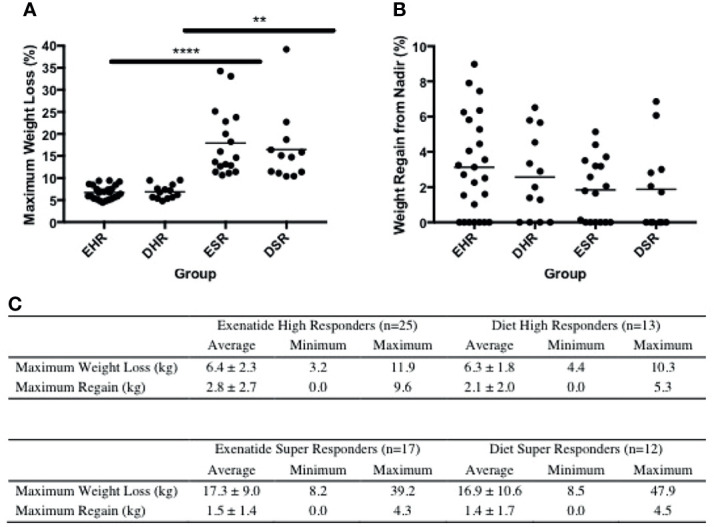
**(A)** Maximum weight loss (%) among high responders and super responders in both treatment groups **** = p < 0.0005, ** = p < 0.005 **(B)** Percentage weight regain from lowest weight achieved among high responders and super responders in both treatment groups. **(C)** Maximum weight loss and regain from lowest weight achieved in exenatide and diet high responder and super responder groups. Values are mean +/- SD. EHR, exenatide high responder; ESR, exenatide super responder; DHR, diet high responder; DSR, diet super responder.

Peak absolute and average percentage weight loss were similar between exenatide and diet high responders (exenatide 11.65 kg, 6.8%, diet 11.91 kg, 6.9% **Figures 3A, C**), however the range of maximum weight loss was larger in the diet high responder group (2.90kg – 47.90kg) compared to the exenatide high responder group (4.20kg – 39.20kg). Super responders accounted for 23% of individuals treated with exenatide and 36% of those treated with diet. This difference was not significant (p = 0.55, **Figure 1A**), nor was there a significant difference in maximum percent weight loss between exenatide and diet super responders (p = 0.62). Super responders had higher peak weight loss compared to high responders in both treatment groups (**Figure 3A**). Super responders demonstrated more than twice as much weight change at 3 months compared to responders, and also achieved more weight loss overall (**Figure 3C**).

We observed weight regain from peak loss during the treatment period among high and super responders in both treatment groups (**Figures 3B, C**). The average weight regain in the exenatide high responder group and the diet high responder group was not significantly different (2.8 +/- 2.7kg *vs* 2.1 +/- 2.0kg, **Figures 3B, C**).

### Metabolic Parameters

For the study population as a whole, there was a positive correlation between baseline body weight and insulin levels (r=0.43, p < 0.001), waist circumference (r=0.35, p < 0.0014) and CRP levels (r=0.45, p < 0.001), however there was no correlation between baseline insulin levels and percentage weight loss (r=0.14, p = 0.2). Both exenatide and diet high responders showed significant reductions in waist circumference, resting energy expenditure, and triglycerides (**Table 2**). Diet, but not exenatide, high responders also showed significant reductions in insulin levels, systolic blood pressure, and percent body fat (**Table 2**). Exenatide high and low responders both showed a significant reduction in total cholesterol and LDL cholesterol, while only diet high responders showed similar reductions in these lipid parameters (**Table 2**). HDL did not increase in either the exenatide or the diet high responder groups (**Table 2**).

### Predictors of Responder and Super Responder Status

We examined several possible physiologic predictors of at least 5% weight loss response to either exenatide or diet. Baseline age, weight, BMI, waist circumference, total cholesterol, and resting energy expenditure were all tested as variables that might predict responder status using multivariable logistic regression. None of these variables served as predictors for high responder status in either treatment group (**Table S2A**). Super responders were very slightly older and had larger waist circumferences compared to high responders (**Table S2B**). In both treatment groups, the magnitude of weight loss by 12 weeks predicted super responder status (exenatide *p=*0.0080, diet *p*=0.0098).

### Metabolomics Analysis

**Figure 4:**
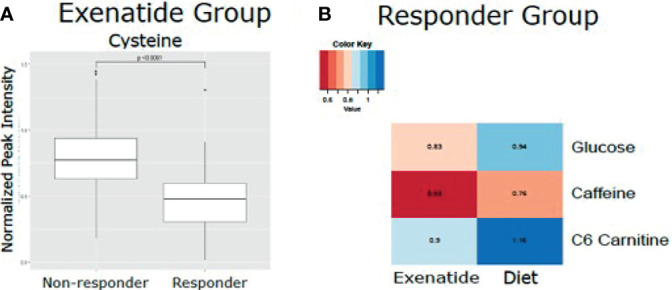
**(A)** Mean normalized LC-MS peak intensity for cysteine, the only metabolite that was statistically different based on Wilcoxon Rank Sum test, between non-responder and responders in the Exenatide treated group. **(B)** Heatmap depicting the differences in metabolite levels, as measured by LC-MS peak intensity, that were statistically different (p<0.05) by Wilcoxon Rank Sum test between responders in the Exenatide and Diet treated groups.

**Figure 5:**
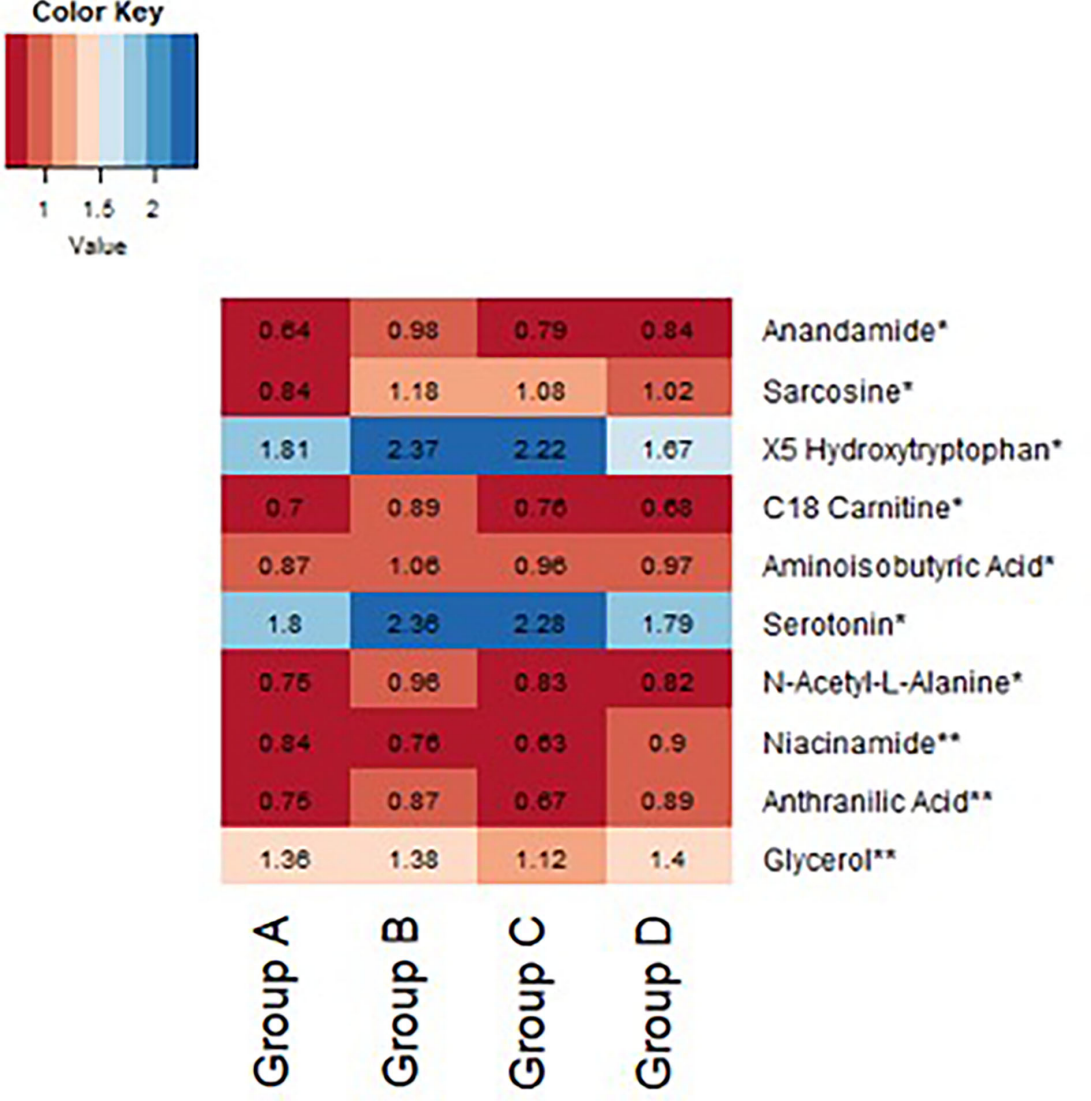
Heatmap demonstrating difference in baseline normalized peak intensities of metabolites between exenatide low responders (Group A, n = 22), exenatide high responders (Group B, n = 14), diet low responders (Group C, n = 11), and diet high responders (Group D, n = 12). *Metabolite peak intensities statistically significant between Group A and B based on Wilcoxon Rank Sum tests (p<0.05). **Metabolite peak intensities statistically significant between Group C and D based on Wilcoxon Rank Sum tests (p < 0.05).

In an exploratory analysis, we sought to identify metabolites that were associated with exenatide treatment response by leveraging a targeted LC-MS method that measures known metabolites (17, 18). When comparing exenatide responders to exenatide non-responders, we found that responders had significantly lower baseline cysteine levels compared to non-responders (*p <*0.0001, **Figure 4A**). Next, we compared exenatide responders to diet responders and found higher baseline glucose, caffeine, and C6 carnitine (hexanoylcarnitine) levels in exenatide responders (*p ≤*0.04, **Figure 4B**). Finally, diet high responders exhibited increased niacinamide, anthranilic acid, and glycerol levels at baseline compared to diet low responders (p <0.04, **Figure 5** and **Table S3**), while exenatide high responders had higher anandamide, sarcosine, X5 hydroxytryptophan, C18 carnitine, aminoisobutyric acid, serotonin, and N-acetyl-L-alanine (p <0.05, **Figure 5** and **Table S3**).

### Thermic Effect of Food

The thermic effect of food (TEF) was measured at baseline and after three months of either exenatide or diet treatment. At baseline, there were no differences between the groups (TEF 4.32 +/- 2.1 kcal/kg exenatide, 4.49 +/- 2.0 kcal/kg diet, **Table 2**). After 3 months of treatment, exenatide high and low responders showed a decrease in the thermic effect of food (high responders – 0.96 kcal/kg, p=0.04, low responders – 1.55 kcal/kg, p=0.006, **Table 2**). In contrast, diet high and low responders showed no change in the thermic effect of food (high responders - 0.37 kcal/kg p=0.56, low responders +0.05 p=0.56, **Table 2**).

### Adverse Events

The most common adverse events for the entire study population were nausea (57%), decreased appetite (36%), and headache (26%). There was significantly more nausea among those randomized to exenatide compared to diet/placebo (70% *vs* 25%, p < 0.0001). Decreased appetite was also significantly more common in the exenatide group (41% compared to 24%, p =0.03). Headache was not significantly different between the two groups (27% exenatide, 24% diet, p=0.66). Among high responders, reports of decreased appetite were not significantly different between exenatide and diet/placebo groups (p=0.86). The incidence of nausea, decreased appetite, and headache decreased throughout the study in both treatment groups.

### Dropouts

We observed a 40% dropout rate (this includes subjects who withdrew consent and those who were lost to follow-up) in both treatment groups from the time of randomization to study week 12. Reported reasons for dropping out of the study are shown in **Figure S2A** by treatment group. At week 12, subjects who did not lose at least 5% body weight in either group were discontinued from the study per protocol design, and as such those individuals are considered study completers and not dropouts. After week 12, the dropout rate in both the exenatide and diet groups was lower than expected, 12% in both treatment groups. Most subjects who were lost to follow up or dropped out did so very soon after randomization: 17% of those randomized to exenatide and 18% randomized to diet did not complete the 2-week treatment visit. An additional 25% of those in the exenatide and 22% in the diet group dropped out after 2 weeks of treatment (**Figure S2B**).

## Discussion

Weight loss can result in improvement or even reversal of significant metabolic sequelae of obesity, including type 2 diabetes (16). GLP-1R agonists are associated with significant long-term weight loss and are very rapidly gaining popularity for the treatment of obesity (10–16). Clinicians’ experiences confirm what has been reported in large clinical studies: there is a wide range of weight loss responses and maintenance of weight loss with these agents. Understanding the heterogeneity of the weight loss response to specific pharmacotherapeutics, as well as hypocaloric diet, has tremendously important clinical and public health implications. The present study examines patterns of weight loss associated with twice daily exenatide treatment in overweight and obese women without diabetes.

In this randomized, single-blind study, we observed similar average weight loss with 6 months of treatment with twice-daily exenatide compared to diet and matched placebo injections. High responder (≥ 5% weight loss) and super high responder (≥ 10% weight loss) rates were also similar across treatment groups, however there was a trend toward a larger percentage of both high and super high responders in the diet group. The greatest amount of weight loss achieved in the entire study population was in the diet group, and there was also less weight regain in this group. To put the present study in context, O’Neil et al. reported the results of a phase 2 randomized, placebo and active controlled double blind dose ranging study looking at weight loss with semaglutide *vs* liraglutide *vs* placebo in individuals with obesity (13). In this study, participants received monthly nutrition counseling recommending 500 kcal/day deficit calculated based on REE, similar to the counseling provided to the diet group in the present study, and performed daily subcutaneous injections of study treatment or placebo. In terms of responder status, 60-91% of those who received semaglutide 0.05-0.4mg daily, 72% of those who received liraglutide, and 23% of those who received placebo lost 5% body weight (13). Looking at 10% weight loss response, 21-74% of those who received increasing doses of semaglutide, 41% of those who received liraglutide, and 10% of those who received placebo lost achieved this degree of weight loss. More recently, the semaglutide STEP 3 study, which compared 2.4mg of once weekly semaglutide to placebo and included intensive behavioral therapy in both groups (hypocaloric diet plus exercise), reported 5% weight loss in 87% of those treated with semaglutide and 48% of those treated with placebo lost 5% body weight, and 10% weight loss in 75.3% treated with semaglutide and 27% treated with placebo (14). In the present study 56% of exenatide and 76% of diet/placebo treated individuals lost ≥ 5% body weight and 43% of exenatide treated and 55% of diet treated individuals lost ≥ 10% body weight–this is a much higher response rate in the placebo group compared to studies with similar nutrition counseling and use of GLP1RA. As we did not offer nutrition counseling to the exenatide group, our study design may have underestimated the weight loss benefit of exenatide; in clinical practice, medications prescribed for weight loss are used in conjunction with lifestyle modification include changes to diet and exercise. A variable weight loss response to exenatide treatment was apparent early on, with greater initial weight loss at 3 months predicting super high responder status. This finding is consistent with our previous study showing that the differential response to exenatide is evident early on during the treatment course (11). Similar results have been seen with liraglutide (19).

The much higher placebo response in the present study could have several explanations. Twice daily pre-prandial injections served to blind subjects to study treatment in the present study, which may have made those in the hypocaloric diet group more invested than they would have been if they had known they were receiving only dietary counseling and not active pharmacotherapy. Additionally, the process of performing a subcutaneous injection and then waiting 15-30 minutes before eating is similar to a practiced mindfulness intervention. Mindful eating, which includes a deliberate pause before beginning a meal. has been shown to reduce the quantity of food eaten (20). The current study design unfortunately cannot tease apart the effects of injections followed by a pause before eating and hypocaloric diet itself in the control group, however the behavior and psychology of twice daily pre meal injections may be an important explanation for the high percentage of responders and super responders in the hypocaloric diet group. Although not statistically significant, the diet group may have had less weight regain because these individuals made ongoing changes to food intake—quality and/or quantity–that were more durable than the effect of exenatide. There is also a treatment plateau (or escape from efficacy) that is seen with any type of pharmacotherapy for weight loss. Future studies might compare weight loss with GLP1RA treatment with and without nutrition counseling in order to better isolate the pharmacotherapeutic effect of GLP1RA on body weight, and other metabolic parameters.

The thermic effect of food (TEF), which comprises approximately 8-10% of total daily energy expenditure, is typically unaffected by weight loss (21). In this study, 12 weeks of exenatide treatment led to a significant decreased in TEF in both the high and low responder groups, while TEF was unchanged in the diet group. This suggests that the reduction in TEF is specifically attributable to exenatide, perhaps due to delayed gastric emptying, rather than weight loss. Previous studies have similarly shown that treatment with GLP1RA leads to a decrease in TEF (22, 23). The compensatory metabolic mechanisms that favor weight loss despite the decrease in TEF among exenatide high responders remain unknown but are an important area of future research.

Exenatide treatment led to reductions in total and LDL cholesterol levels among both high and low weight loss responders, while these lipid reductions were seen in only the diet high responder group. This finding in the present study may be explained by the fact that even low responders to exenatide achieved modest weight loss, and/or there is a specific cholesterol lowering mechanism related to GLP1R agonism that is separate from weight loss. Among exenatide responders, total cholesterol decreased by 9.6%, LDL by 7.8%, and HDL by 13%; among diet responders, total cholesterol decreased by 7.2%, LDL by 1.7% (nonsignificant) and HDL by 11.6%. The decrease in HDL is therefore proportional to the decrease in total cholesterol in both groups. Additionally, in the semaglutide STEP 3 study, Wadden et al. reported that among lipid parameters, HDL did not change significantly in the semaglutide treatment group compared to placebo group (14). Weight loss, with or without the use of pharmacotherapy, is often associated with a decrease in total cholesterol and LDL but not always associated with an increase in HDL. Interventions more likely to increase HDL specifically include exercise, modest alcohol consumption, increased consumption of healthy fats, and smoking cessation. Additionally, the present study did not show a significant change in fasting triglycerides among those treated with exenatide, including high responders, although several human and rodent studies have reported significant decreases in triglycerides following treatment with GLP1RA (24, 25).

Diet but not exenatide responders had lower insulin levels after 12 weeks of treatment, which is consistent with greater reduction in body fat in the diet but not exenatide responder group. This study did not encourage any change in baseline physical activity levels, however physical activity was not monitored rigorously so it is possible that the diet responders were more physically active throughout the treatment period than the exenatide responders and as such had greater reduction in insulin levels with similar weight loss. This finding is consistent with the finding that liraglutide treatment was associated with significant weight loss but not improvement in HOMA-IR among individuals with CAD and newly diagnosed T2DM (26).

In exploratory analyses leveraging a targeted metabolomics platform, we found an association of significantly lower baseline circulating cysteine concentrations with weight loss following exenatide treatment. Cysteine is an organosulfur compound that plays important roles in many enzymatic reactions in the body including redox reactions, metal coordination, and structural disulfide formation. It is also the precursor to the antioxidant glutathione. In epidemiology studies, total cysteine levels have been positively correlated with BMI and obesity (27, 28). Moreover, modulating the levels of cysteine has been demonstrated to affect appetite and weight in rodent models (29).

We also found a trend toward higher baseline levels of serotonin, aminoisobutyric acid, anandamide, and sarcosine in exenatide super responders. Anandamide is a lipid metabolite and neurotransmitter that plays a role in feeding behavior and obesity (17, 30). Aminoisobutyric acid is known to increase fatty acid oxidation and to decrease fat mass (31). Notably, aminoisobutryric acid increases following exercise and induces browning of white fat (32). In sum, the metabolite findings in our sub study point toward metabolite pathways that may impact biological pathways related to the weight loss, however future studies in a larger cohort are required to validate these findings. We acknowledge that a limitation of the metabolomics analysis is our small sample size which meant we were underpowered to definitively determine differences in metabolite concentration associations with treatment effect and our results could be biased by outliers.

The most common adverse events of nausea and decreased appetite were more prevalent in the exenatide group than the diet group, and more high responders to exenatide experienced nausea than low responders. Nausea itself may have contributed to the higher weight loss among exenatide responders, however as the study progressed, the incidence of side effects diminished in both exenatide groups.

In the field of obesity medicine, it is of great interest to identify predictors of weight loss in order personalize pharmacologic and lifestyle interventions. For example, a recent study examined predictors of A1C reduction with exenatide treatment in individuals with type 2 diabetes and found that elevated initial A1C was the strongest predictor of magnitude of A1C reduction (33). In our study, we found that greater weight loss early in the treatment period predicted super responder status in both treatment groups, and the direction of the effect of baseline weight suggests that individuals with higher starting BMIs have a higher likelihood of reaching super responder status, similar to the effect of initial A1C on glycemic improvement. Specifically, for every additional 1kg in baseline weight, the odds of being super responder is increased by.7% (odds ratio of 1.007). Larger studies will be better suited to examine additional predictors parameters, including genetic factors, that could contribute to a variable weight loss response.

This study has several limitations. First, all of the subjects in this study were relatively healthy women with overweight or obesity without severe metabolic complications, which limits the generalizability of these findings to a more heterogeneous population with obesity. Future studies should include men and individuals with more poorly compensated obesity. Additionally, pharmacotherapies that are approved for weight loss are meant to be used only in combination with lifestyle modification, and in this study we did not include lifestyle modification with exenatide treatment. Our study design therefore does not reflect the clinical use of exenatide in combination with dietary change. Another important limitation is that the present study did not require subjects to maintain detailed food logs, so we are unable to determine whether there were proportional differences in macronutrient intake in the exenatide and hypocaloric diet groups. Consequently, we cannot assess whether the hypocaloric diet group consumed less protein or more cysteine rich food, which would affect the changes we found in lean mass and metabolite levels respectively.

## Conclusion

The present study examined extended patterns of weight loss in overweight and obese women who demonstrated an early robust response to either twice daily exenatide or hypocaloric diet. After attaining at least 5% weight loss at 12 weeks, our responder population demonstrated a highly variable longer term response to both exenatide and diet. The magnitude of early weight was found to be a predictor of super responder status in both the exenatide and diet treatment groups. Our study suggests that while exenatide may have a few specific metabolic benefits, weight loss with this agent is similar to that achieved with a hypocaloric diet. Larger studies are needed to examine more carefully the question of weight regain with hypocaloric diet and regular nutrition counseling compared to treatment with this, or other, GLP1R agonists.

## Data Availability Statement

The raw data supporting the conclusions of this article will be made available by the authors, without undue reservation.

## Ethics Statement

The studies involving human participants were reviewed and approved by Beth Israel Deaconess Medical Center Institutional Review Board. The patients/participants provided their written informed consent to participate in this study.

## Author Contributions

All of the authors contributed significantly to one or more of: data collection, data analysis, manuscript preparation.

## Funding

The authors declare that this study received funding from Astra Zeneca. The funder provided exenatide and financial support. The funder was not involved in the study design, collection, analysis, and interpretation of data, the writing of this article or the decision to submit it for publication. This work was conducted with support from Harvard Catalyst | The Harvard Clinical and Translational Science Center (National Center for Advancing Translational Sciences, National Institutes of Health Award UL 1TR002541) and financial contributions from Harvard University and its affiliated academic healthcare centers. The content is solely the responsibility of the authors and does not necessarily represent the official views of Harvard Catalyst, Harvard University and its affiliated academic healthcare centers, or the National Institutes of Health.

## Conflict of Interest

The authors declare that the research was conducted in the absence of any commercial or financial relationships that could be construed as a potential conflict of interest.

## Publisher’s Note

All claims expressed in this article are solely those of the authors and do not necessarily represent those of their affiliated organizations, or those of the publisher, the editors and the reviewers. Any product that may be evaluated in this article, or claim that may be made by its manufacturer, is not guaranteed or endorsed by the publisher.
