# Supplementary material for: Weight Loss Outcomes Among Early High Responders to Exenatide Treatment: A Randomized, Placebo Controlled Study in Overweight and Obese Women
Source: Front Endocrinol (Lausanne). 2021 Nov 17;12:742873. doi: 10.3389/fendo.2021.742873 (PMC8635796; doi:10.3389/fendo.2021.742873)
Supplement: Supplementary file 1 [file DataSheet_1.pdf]

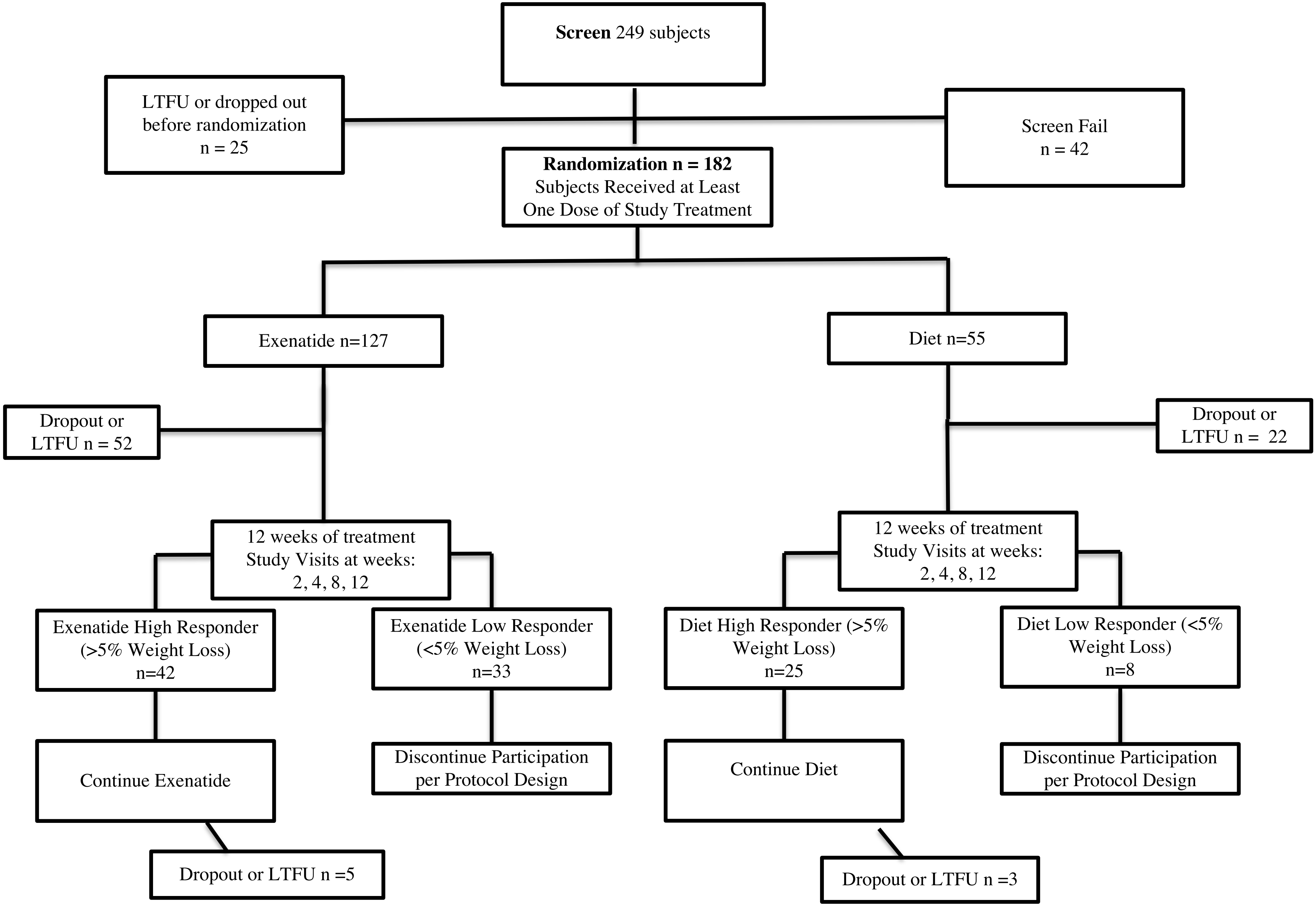

Figure S1: Study design.

	ER (n=42)	DR(n=25)	p
Age (years)	43.9 ± 12.1	44.8 ± 12.9	0.77
BMI (kg/m^2)	35.8 ± 5.4	35.0 ± 5.3	0.55
Systolic BP (mmHg)	125.0 ± 11.0	124.8 ± 13.1	0.92
Diastolic BP (mmHg)	73.3 ± 9.2	74.5 ± 12.3	0.67
Heart Rate (bpm)	68.0 ± 11.9	64.9 ± 12.2	0.30
Total Cholesterol (mg/dL)	196.8 ± 34.3	196.6 ± 31.5	0.97
HDL Cholesterol (mg/dL)	61.0 ± 19.3	60.0 ± 11.1	0.79
LDL Cholesterol (mg/dL)	115.6 ± 30.8	114.8 ± 26.7	0.92
Triglycerides (mg/dL)	111.0 ± 61.5	108.7 ± 53.7	0.88
Hemoglobin A1c (%)	5.6 ± 0.3	5.6 ± 0.3	0.79
Waist Circumference (cm)	110.0 ± 11.5	111.5 ± 12.4	0.61
Resting Energy Expenditure (kCal/d)	1548.9 ± 224.4	1542.5 ± 194.6	0.91
Percent Body Fat (%)	44.0 ± 4.4	43.8 ± 3.8	0.87

Table S1: Baseline characteristics of responders in both treatment groups (n=67). ER = exenatide responder. DR = diet responder

A

	Odds Ratio	Confidence Interval
Age (years)	1.03	0.97 – 1.09
Weight (kg)	0.98	0.98 – 1.20
BMI (kg/m^2)	0.75	0.56 – 1.01
Waist Circumference (cm)	1.05	0.96 – 1.13
Total Cholesterol (mg/dL)	0.99	0.97 – 1.01
Resting Energy Expenditure (kCal/d)	1.16	0.94 – 1.42

B

	Adjusted Odds Ratio	Confidence Interval
Age (years)	1.01	0.98 – 1.05
Weight (kg)	1.007	0.98 – 1.04
Waist Circumference (cm)	2.00	0.84 – 4.83
Total Cholesterol (mg/dL)	1.00	0.99 – 1.02

Table S2: (A) Metabolic predictors of high responder status for both treatment groups (B) Metabolic predictors of super responder status for both treatment groups.

Table S3. Differences in baseline metabolite levels in responders compared to super responders within treatment groups.

Exenatide Treatment Group			
Metabolite	Responder (n = 22)	Super Responder (n =14)	P
	Mean (SD)	Mean (SD)	
Anandamide	0.64 (0.20)	0.98 (0.35)	0.001
Sarcosine	0.84 (0.29)	1.18 (0.27)	0.002
X5 Hydroxytryptophan	1.81 (0.86)	2.37 (0.77)	0.01
C18 Carnitine	0.70 (0.25)	0.89 (0.22)	0.02
Aminoisobutyric Acid	0.87 (0.30)	1.06 (0.21)	0.02
Serotonin	1.80 (0.83)	2.36 (0.77)	0.02
N-Acetyl-L-Alanine	0.75 (0.26)	0.96 (0.26)	0.04
Diet Treated Group			
Metabolite	Responder (n = 11)	Super Responder (n = 12)	P
	Mean (SD)	Mean (SD)	
Niacinamide	0.63 (0.18)	0.90 (0.25)	0.02
Anthranilic Acid	0.67 (0.13)	0.89 (0.27)	0.02
Glycerol	1.12 (0.25)	1.40 (0.32)	0.02

Data is presented as mean (standard deviation) LC-MS spectra peak intensity after normalization to pooled plasma for each metabolite in Exenatide treated responders (n = 22) compared to Exenatide super responders (n = 14) and diet treated responders (n = 11) compared to placebo super responders (n = 12). P values were calculated using Wilcoxon rank sum on log transformed peak areas.

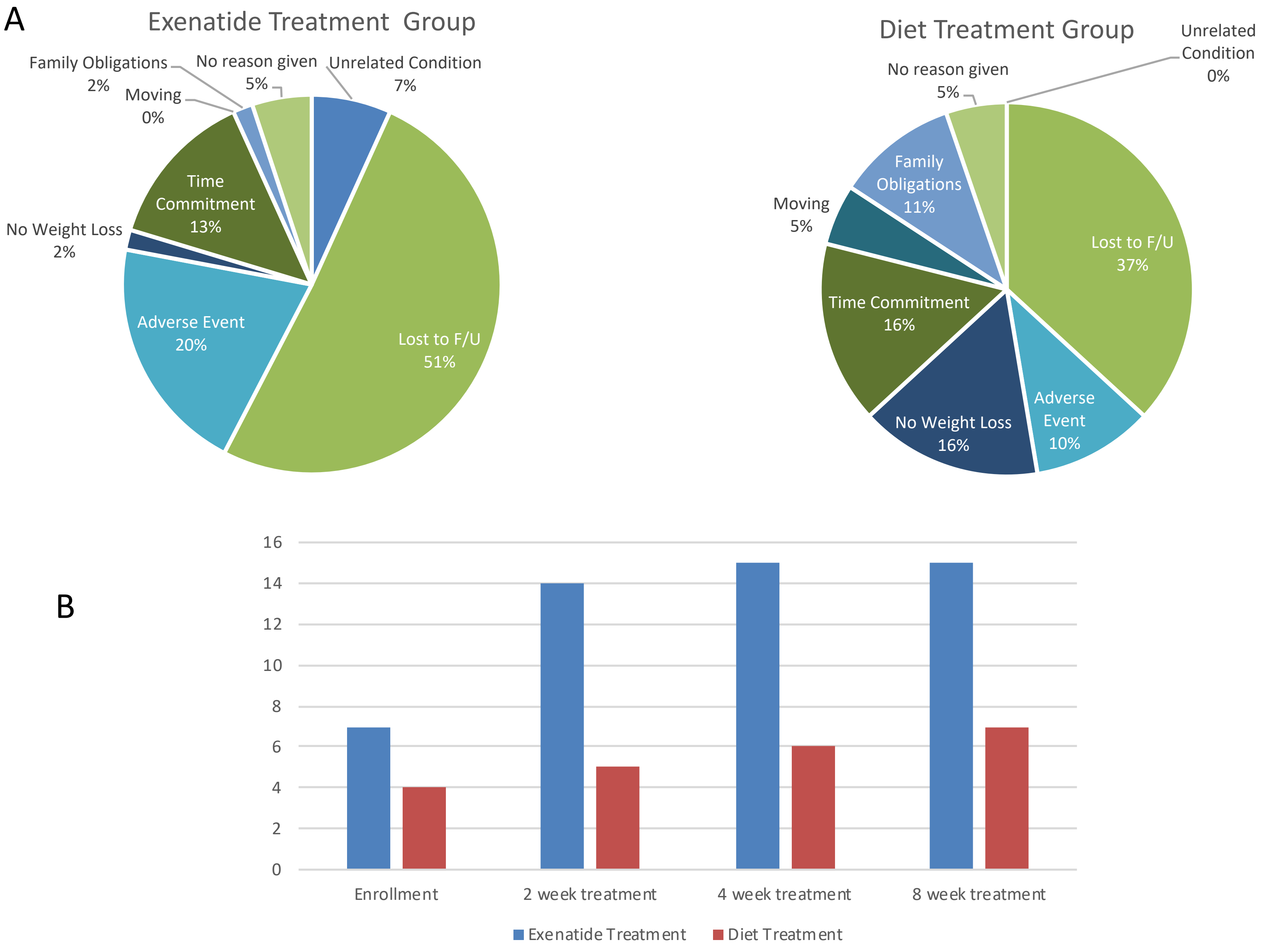

Figure S2: (A) Dropout by Treatement Group (B) Dropout by Study Visit
